# The effects of the histone deacetylase inhibitor 4-phenylbutyrate on gap junction conductance and permeability

**DOI:** 10.3389/fphar.2013.00111

**Published:** 2013-09-03

**Authors:** Joshua Kaufman, Chris Gordon, Roberto Bergamaschi, Hong Z. Wang, Ira S. Cohen, Virginijus Valiunas, Peter R. Brink

**Affiliations:** Department of Physiology and Biophysics, Stony Brook UniversityStony Brook, NY, USA

**Keywords:** connexin43, 4-phenylbutyrate, gap junction, conductance, permeability

## Abstract

Longitudinal resistance is a key factor in determining cardiac action potential propagation. Action potential conduction velocity has been shown to be proportional to the square root of longitudinal resistance. A major determinant of longitudinal resistance in myocardium is the gap junction channel, comprised connexin proteins. Within the ventricular myocardium connexin43 (Cx43) is the dominantly expressed connexin. Reduced numbers of gap junction channels will result in an increase in longitudinal resistance creating the possibility of slowed conduction velocity while increased numbers of channels would potentially result in an increase in conduction velocity. We sought to determine if inhibition of histone deacetylase (HDAC) by 4-phenylbutyrate (4-PB), a known inhibitor of HDAC resulted in an increase in junctional conductance and permeability, which is not the result of changes in single channel unitary conductance. These experiments were performed using HEK-293 cells and HeLa cells stably transfected with Cx43. Following treatment with increasing concentrations of 4-PB up-regulation of Cx43 was observed via Western blot analysis. Junctional (*g*_j_) conductance and unitary single channel conductance were measured via whole-cell patch clamp. In addition intercellular transfer of lucifer yellow (LY) was determined by fluorescence microscopy. The data in this study indicate that 4-PB is able to enhance functional Cx43 gap junction coupling as indicated by LY dye transfer and multichannel and single channel data along with Western blot analysis. As a corollary, pharmacological agents such as 4-PB have the potential, by increasing intercellular coupling, to reduce the effect of ischemia. It remains to be seen whether drugs like 4-PB will be effective in preventing cardiac maladies.

## INTRODUCTION

In multicellular organisms, the direct communication between adjacent cells is mediated via protein structures known as gap junctions (Sohl and Willecke, 2004). Connexins belong to a family of integral membrane proteins that form the underlying structure of gap junctions (Pointis, 2006). Various compounds including metabolites, ions, and fluorescent dyes can be exchanged from cell to cell via passive diffusion through these protein channels. Gap junctions consist of two smaller hemichannels called connexons, which are each composed of six smaller subunits called connexins. These protein channels are responsible for coordinating cellular activity in most biological systems including the myocardium, brain, and vascular endothelium.

The present study is focused on the role of connexin43 (Cx43) found predominantly in ventricular myocardium. In the myocardium, gap junctions create an electrical conduit between adjacent cells that is vital to normal cardiac function. The result is a synchronized propagating action potential and subsequent muscle contraction that starts in the sinoatrial (SA) node and ends within the cells of the ventricular myocardium. The conduction velocity of the cardiac action potential is linked to the longitudinal resistance arising from cytoplasm and gap junctional membranes (Rudy, 2001; Donahue and Laurita, 2011) where conduction velocity, θ, is inversely proportional to the square root of longitudinal resistance, *R*_i_ or (θ ∝ 1/√*R*_i_ ; Hodgkin and Huxley, 1952).

Under pathological conditions such as cardiac ischemia rapid changes in ionic homeostasis often cause irreparable cellular damage that can affect gap junction distribution within myocytes (Saffitz et al., 2007). Ischemia also induced uncoupling in myocytes and has been linked to the induction of reentrant currents seen during ventricular tachycardia (VT; Xing et al., 2003).

4-Phenylbutyrate (4-PB) belongs to a group of agents known as histone deacetylase (HDAC) inhibitors, and is currently used as a promising anti-cancer drug (Khan et al., 2007). In previous studies, the drug 4-PB has been reported to increase Cx43 expression (Asklund et al., 2004; Khan et al., 2007). HDACs are able to induce histone hyper-acetylation, altered chromatin structure, and modulations in gene expression that allow for increased mRNA transcription. By causing DNA to lose its affinity to histone proteins, exposure of portions of DNA allow for increased mRNA transcription that codes for increased Cx43 translation.

The aim of this study was to investigate the role of 4-PB with regards to its ability to induce increases in junctional conductance and correlate it with up-regulation of Cx43. It was hypothesized that those cells that were exposed to 4-PB would express increased levels of Cx43, as previously reported (Asklund et al., 2004; Khan et al., 2007) and result in an increased number of gap junction channels. Following treatment with increasing concentrations of 4-PB, up-regulation of Cx43 was observed via Western blot analysis consistent with previous studies (Asklund et al., 2004; Khan et al., 2007). Junctional conductance (*g*_j_) and intercellular transfer of lucifer yellow (LY) were measured via the whole cell patch clamp and by fluorescence microscopy. Demonstration that 4-PB can increase gap junction conductance and reduce longitudinal resistance allows for its potential use as an agent to reduce life-threatening conduction abnormalities, including reentrant ventricular arrhythmias.

## MATERIALS AND METHODS

### CELLS AND CULTURE CONDITIONS

Experiments were performed on HeLa cells stably transfected with mCx43 or HEK-293 cells that were endogenously expressing Cx43. Production and characterization of these cells, culture conditions, and staining methods for identification of specific cells have been described previously (Valiunas et al., 2000, 2001, 2004; Gemel et al., 2004). Experimental groups of cells were cultured with a medium containing 5 mM of 4-PB. Both the control and experimental groups of cells were plated onto glass coverslips 1–3 days prior to experimentation and were stored in a CO_2_ incubator (5% CO_2_, 95% ambient air at 37°C) Electrophysiological measurements and dye flux studies were carried out on cell pairs and linear arrays triplets, respectively.

### ELECTROPHYSIOLOGICAL MEASUREMENTS

These experiments were preformed on HEK-293 and HeLa Cx43 cell pairs. A dual voltage clamp method and whole cell recording were used to control the membrane potential of both cells and to measure currents. For electrical recordings, glass coverslips with adherent cells were transferred to an experimental chamber mounted on the stage of an inverted microscope (Olympus IMT-2) equipped with epi-fluorescence imaging. The chamber was perfused at room temperature (RT; 22°C) with bath solution containing (in mM) NaCl, 150; KCl, 10; CaCl_2_, 2; HEPES, 5 (pH 7.4); glucose, 5; 2 mM CsCl and BaCl_2_ were added. The patch pipettes were filled with solution containing (in mM) K^+^ aspartate^-^, 120; NaCl, 10; MgATP, 3; HEPES, 5 (pH 7.2); EGTA, 10 (*p*Ca ~ 8); filtered through 0.22-μm pores. Patch pipettes were pulled from glass capillaries (code GC150F-10; Harvard Apparatus) with a horizontal puller (Sutter Instruments).

### DYE FLUX STUDIES

Experiments were performed on HeLa Cx43 cell triplets that were linearly coupled, using the direct dye injection technique. LY (Molecular Probes) was dissolved in the pipette solution to reach a concentration of 2 mmol/L. The donor cell was attached to a patch pipette connected to a micromanipulator (Narishige International), and an amplifier (Axopatch 200B) so that the membrane potential could be observed to help obtain the whole cell patch. The use of the whole cell patch clamp was used to ensure delivery of dye intracellularly without leakage into the bathing solution. Fluorescent dye cell-to-cell spread was monitored using the digital charge-coupled diode (CCD) camera PixelFly (12-bit; The Cooke). LY concentration is directly proportional to fluorescence intensity. A picture of the cell triplet’s fluorescence was taken every 60 s over a period of 10 min (CamWare v2.10). Fluorescence intensity of each cell was measured and recorded every minute. The background intensity was subtracted from the respective images.

### WESTERN BLOT

HEK-293 cells were collected from 35 mm plates by scraping. Cell suspensions were centrifuged at 14000 rpm at RT for 5 min (calculated *g* = 13148), supernatants were removed, and the pellets were re-suspended in cold 1× phosphate-buffered saline (PBS). The pellets were centrifuged, supernatants removed, pellets then re-suspended in cold radio-immnuoprecipitation assay (RIPA) buffer (R0278, Sigma), protease inhibitor cocktail (AEBSF, aprotinin, bestatin hydrochloride, E-64, EDTA, leupeptin; P2714, Sigma), sodium orthovanadate (S-6508, Sigma), and PMSF (P-7626, Sigma). Samples were then centrifuged at 4°C, 14000 rpm (*g* = 13148) for 10 min, supernatants were transferred to pre-chilled microtubes. Protein concentration of each sample was determined by the Bradford assay. Volumes containing 30 μg of total protein of each lysate were mixed with equal volumes of Laemmli sample buffer (161-0737, Bio-Rad) containing β-mercaptoethanol and boiled for 5 min at 95°C. All samples were centrifuged for 1 min at 14000 rpm (*g* = 13148) at RT before being loaded on a SDS-polacrylamide gel (4% stacking gel, 10% separating gel). Prestained Protein Ladder (SM0671, Fermentas) or MagicMark XP Protein Standard (LC5602, Invitrogen) was loaded along with the samples. After separation by electrophoresis, proteins were transferred to Immobilon-P membrane (Millipore) by electrophoresis in tris–glycine/methanol buffer. Non-specific antibody binding was blocked for 1 h at RT in 5% Blotting Grade Blocker non-fat dry milk (Bio-Rad) dissolved in 1× TBST (mixture of Tris-buffered saline and Tween 20). A 43-kDa protein was probed for by incubating the membrane with the Anti-Connexin 43 antibody (C 6219, Sigma) at 1:8000 in 1% milk for 1 h at RT. After washing the membrane well the membrane was incubated with goat anti-rabbit IgG-HRP (sc-2004, Santa Cruz) at 1:10000 in 1% milk. After washing the secondary antibody was detected using SuperSignal West Femto Maximum Sensitivity Substrate (34095, Pierce) and images obtained by exposing the membrane to HyBlot CL Autoradiography Film (E3012, Denville Scientific).

As a loading control for normalization a 55 kDa protein was probed for by incubating the membrane for 1 h at RT with Anti-α Tubulin (sc-8035) at 1:1000 in 1% milk. After washing the membrane well with 1× TBST the membrane was incubated for 1 h at RT with goat anti-mouse IgG-HRP (sc-2005) at 1:10000 in 1% milk. After washing the membrane well with 1× TBST the secondary antibody was detected using SuperSignal West Femto Maximum Sensitivity Substrate (34095, Pierce) and images obtained by exposing the membrane to HyBlot CL Autoradiography Film. ImageJ software (NIH) was used for analysis and quantification of Western blot data.

### SIGNAL RECORDING AND ANALYSIS

Voltage and current signals were recorded using patch clamp amplifiers (Axopatch 200b). The current signals were digitized with a 16-bit A/D-converter (Digidata 1322A; Molecular Devices) and stored with a personal computer. Data acquisition and analysis were preformed with pClamp9 software (Molecular Devices). Curve fitting and statistical analyses were performed using SigmaPlot and SigmaStat, respectively (Jandel Scientific). The Mann–Whitney rank sum test was used for all cases unless otherwise noted, *p* < 0.05 was considered to indicate significant changes. The results are presented as means ± SEM.

## RESULTS

### LY DYE TRANSFER

**FIGURE 1:**
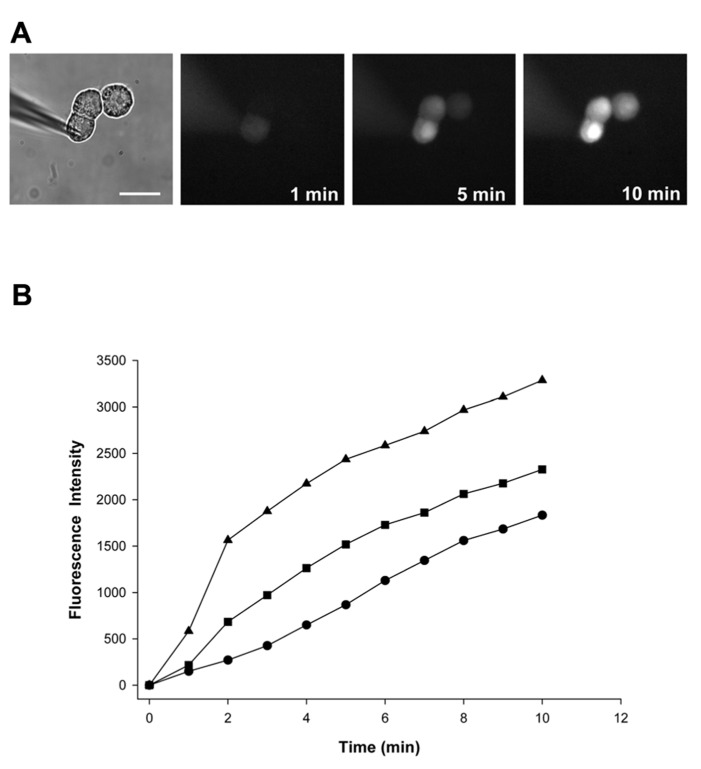
**(A)** A pipette containing 2 mmol/L LY was attached to the cell furthest to the left in whole cell configuration. Epi-fluorescent micrographs were taken at 1, 5, and 10 min after dye injection into the first cell and showed a progressive fluorescence intensity increase in the two adjacent recipient cells. Scale bar: 20 μm. **(B)** Quantification of cell-to-cell spread of LY. Fluorescence intensity plots versus time for the 5 mM 4-PB HeLa Cx43 cell trial shown in **(A)**: first (injected) cell (▲), second cell (■), and third cell (•).

We compared the LY dye spread in control cells and in cells exposed to 4-PB for 48 h. All trials were performed using HeLa Cx43 cell triplets. The amount of LY transfer was determined over time by epi-fluorescence microscopy. One such experiment is depicted in **Figure 1A**. As time elapses, the concentration of LY within a given cell increases due to diffusion of LY from electrode to the first source cell then to adjacent cell through gap junction channels. In this case, three time points are shown (1, 5, and 10 min) after dye injection. Once a linearly coupled cell triplet was identified, the patch pipette containing LY was attached to the donor or source cell in the whole cell mode. Fluorescence intensity was subsequently determined at 1-min time intervals for each experiment in all cells of the triplet. **Figure 1B** shows fluorescence intensity data for the experiment seen in **Figure 1A**.

**FIGURE 2:**
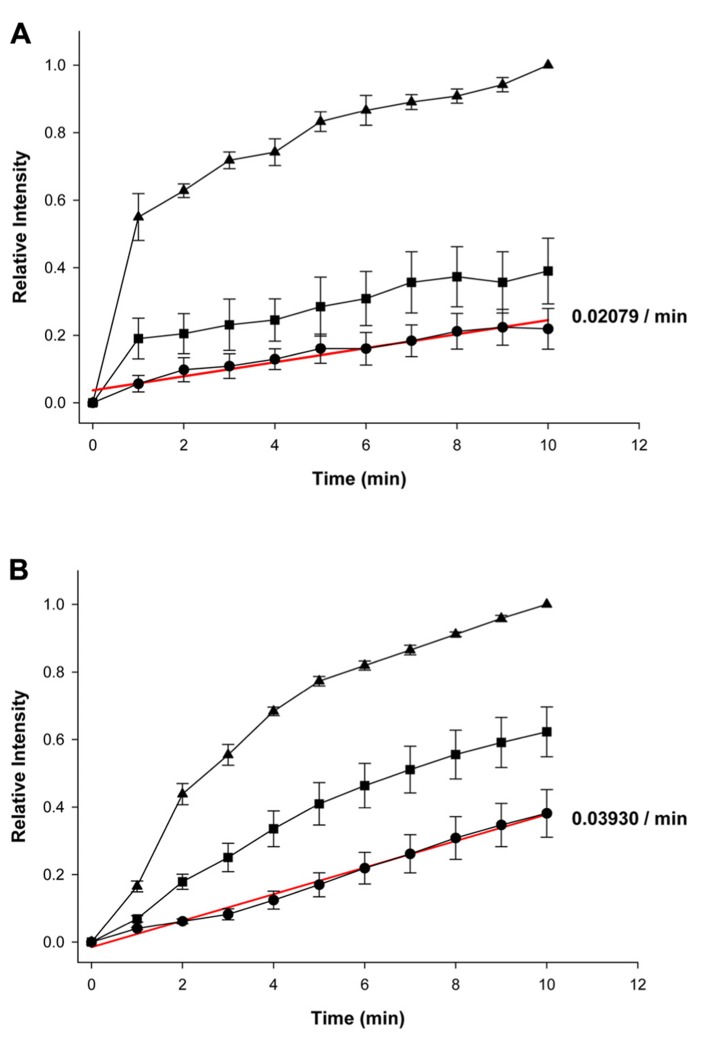
**Summary plots of fluorescence intensity versus time.** **(A)** Summary of averaged and normalized control (*n* = 6 experiments) and **(B)** 5 mM 4-PB data (*n* = 4 experiments). First (source) cell (▲), second cell (■), and third cell (•). Error bars are representative of SE. Solid red lines correspond to the first-order regressions with *r*^2^ = 0.94 and *r*^2^ = 0.99 for control and 4-PB treated cells, respectively.

**Figures 2A, B** summarize the fluorescence intensity data obtained from six experiments in HeLa Cx43 control cells and four experiments with 5 mM 4-PB exposed cells, respectively. This data shows the relative fluorescent intensities for the first (source) cell (▲), second cell (■), and third cell (•). The maximum (steady state) intensity attained in the source cell represents the equivalent of the concentration in the pipette. Fluorescence intensity in each cell increased with time in both the control and 5 mM 4-PB exposed cells. The increases in fluorescence intensity were normalized in both groups of experiments. There was a marked difference, between control and 5 mM 4-PB cells, in dye transfer to the third cell. In control cells, the third cell was able to reach ~21% of the concentration reached by the loading cell compared to the ~39%, which was achieved in the 5 mM 4-PB group (**Figures 2A, B**) in the same time period (10 min).

Because dye transfer between each adjacent cell occurred through Cx43 comprised gap junction channels, comparing the increase in LY fluorescence intensity in the third cell correlates to the amount of Cx43 channels present between adjacent cells. Linear fits of the third cell data to first-order regression (red lines, **Figures 2A, B**) yielded the following slopes: 0.02079 ± 0.001752/min and 0.03930 ± 0.001233/min for control and 4-PB treated cells, respectively. Comparison of the regression lines by analysis of covariance (GraphPad Prism) revealed that the difference between the slopes was significant (*p* < 0.001).

These findings point to an increase of dye permeation upon exposure to 4-PB. Previous studies (Asklund et al., 2004; Khan et al., 2007) have also shown enhanced dye spread with exposure to 4-PB. This can be explained by an increase in the number of functioning channels resulting from increased expression. It is also possible that an increase in junctional conductance might arise from an increase of unitary channel conductance/permeability of Cx43 gap junction channels upon exposure to 5 mM 4-PB. In addition, changes in channel open probability would be predicted to affect junctional conductance. Does 4-PB affect any of these parameters?

### MACROSCOPIC AND UNITARY CONDUCTANCES OF Cx43 GAP JUNCTIONS

**FIGURE 3:**
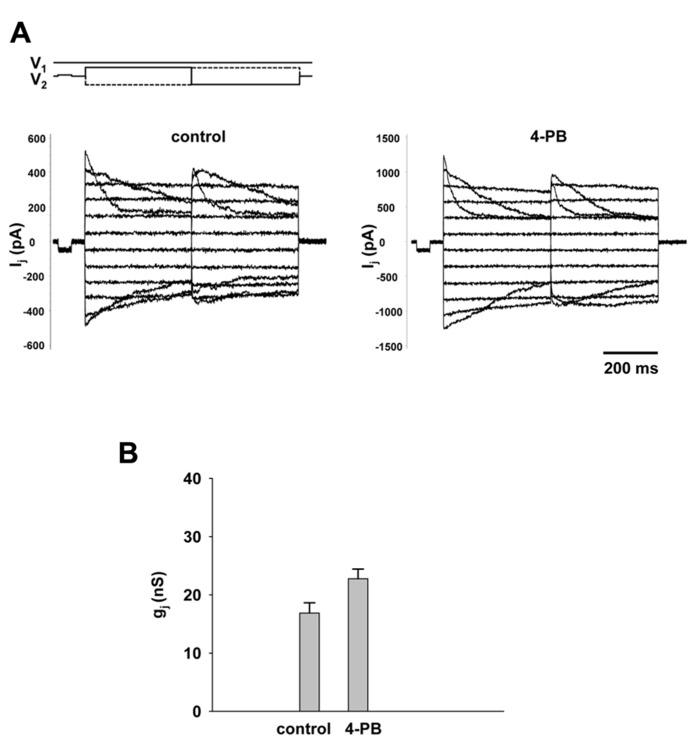
**Macroscopic properties of gap junction channels.** **(A)** Gap junction currents (*I*_j_) elicited from HEK-293 cells by bipolar *V*_j_ pulses (*V*_1_ and *V*_2_; top panel) in control pairs (left panel) and cell pairs with 4-PB exposure (right panel). **(B)** Average of gap junction conductance for 4-PB treated (22.7 ± 1.7 nS, *n* = 31 cell pairs) and control (16.9 ± 1.8 nS, *n* = 32 cell pairs), *p* = 0011.

An important experiment was to determine the effect of 4-PB on macroscopic gap junction conductance. Gap junction currents in HEK-293 cells were recorded using the double whole cell patch clamp. **Figure 3A** shows the voltage protocol (*V*_1_, *V*_2_) and junctional currents recorded from control cells (left panel) and cells treated with 5 mM 4-PB (right panel). Starting from a holding voltage, *V*_h_ of 0 mV, bipolar pulses of 400 ms were delivered to the one cell of a pair to establish *V*_j_ gradient of identical amplitude with either polarity from ±10 to ±110 mV in increments of 20 mV (top panel, **Figure 3A**). In both groups junctional currents exhibited voltage dependent gating typical to Cx43 gap junction channels. The junctional conductances measured in control cells and cells treated with 4-PB are summarized in **Figure 3B**. The average junctional conductances in the control and 5 mM 4-PB groups were 16.9 ± 1.8 nS (*n* = 32) and 22.7 ± 1.7 nS (*n* = 31), respectively. These data indicate a statistically significant (*p* = 0.011) increase in gap junction conductance between these two groups of cells. Previous experiments have suggested that increases in Cx43 expression and number of Cx43 comprised gap junctions are reflected by enhanced gap junctional conductance in double cell patch clamp experiments (Dhein, 2004).

The effect of 4-PB on unitary conductance of Cx43 gap junction channels was determined in selected pairs where only one or two operational channels were observable.

**FIGURE 4:**
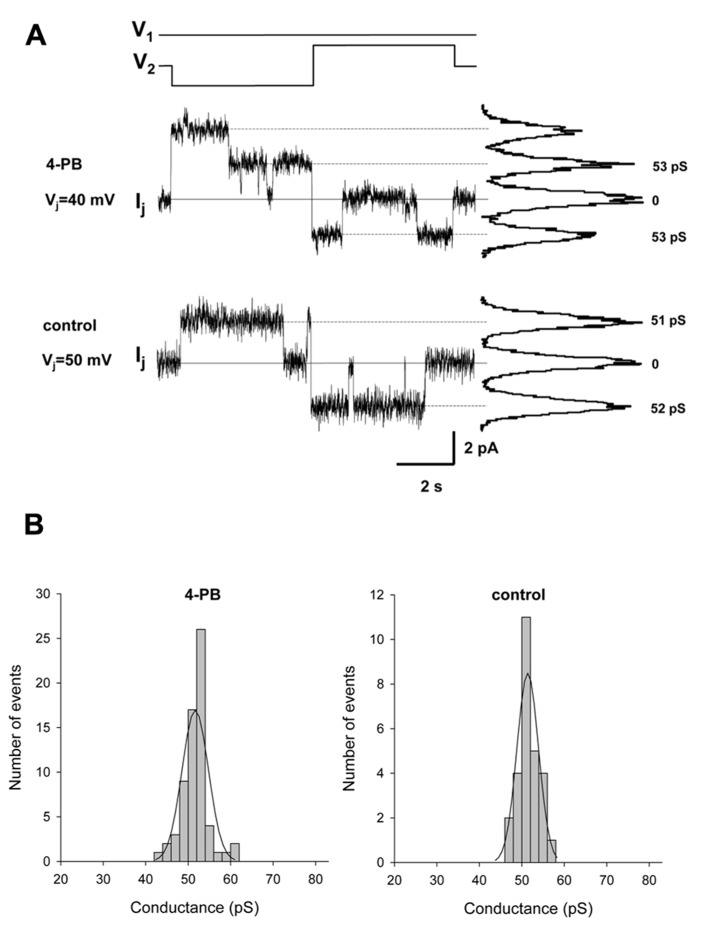
**Single channel properties of gap junction channels.** **(A)** Single channel currents elicited by bipolar *V*_j_ pulses (*V*_1_ and *V*_2_) of 40 mV (top *I*_j_ trace) and 50 mV (middle *I*_j_ trace) in HeLa Cx43 cell pairs with 4-PB exposure and control conditions, respectively. Continuous lines indicate zero current level. The current histograms (right) revealed 53 pS and 51–52 pS conductance levels, for 4-PB exposed and control cells, respectively. **(B)** Histograms of single channel conductances for 4-PB treated (left panel) and control (right panel) cells. The smooth curves represent fits of data to Gaussian distributions. For specific data see text.

The pulse protocol involved an inversion of *V*_j_ polarity but of equal magnitude. **Figure 4A** shows single channel currents recorded from a 4-PB (5 mM) treated HeLa Cx43 cell pair (middle panel) and control HeLa Cx43 cell pair (lower panel). The histograms in **Figure 4B** summarizes the data collected from five HeLa Cx43 cell pairs treated with 5 mM 4-PB (left panel) and four control HeLa Cx43 cell pairs. Both data groups were fitted with a Gaussian (solid lines). The 4-PB treated cells yielded a mean value of 51.7 ± 3.1 pS (*n* = 66) and control cells revealed unitary conductance of 51.4 ± 2.5 pS (*n* = 27), *p* = 0.386. The unitary conductance values correspond to the previously reported Cx43 unitary conductances in 120 mM K^+^ aspartate^-^ solution (Valiunas et al., 2002). These data indicate that 4-PB does not affect the unitary conductance of Cx43 gap junction channels. The long duration open times are similar for the records shown and are consistent with the notion that 4-PB did not significantly affect open probability (Brink et al., 1996).

### WESTERN BLOT ANALYSIS

**FIGURE 5:**
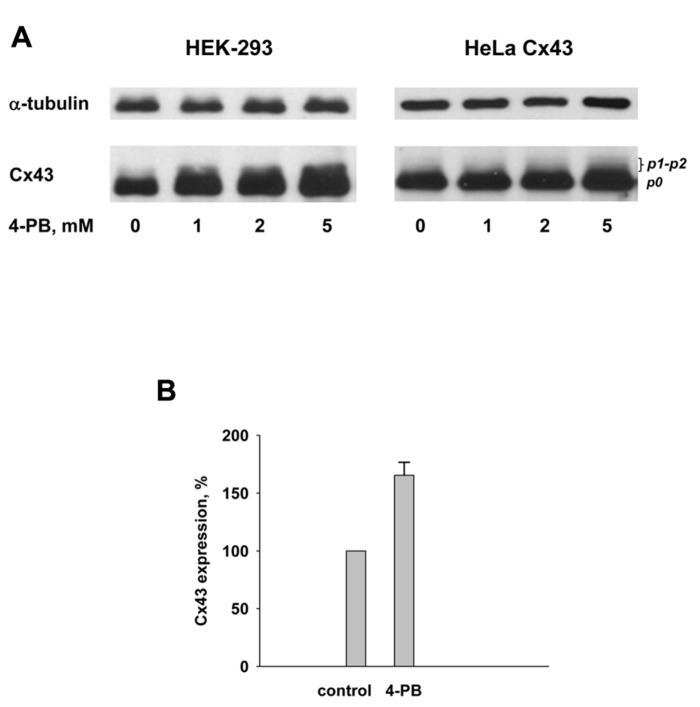
**(A)** Western blot analysis of HEK-293 (left panel) and HeLa Cx43 (right panel) cells using polyclonal Cx43 antibody. Lines from left to right correspond to 0, 1, 2, and 5 mM 4-PB, respectively. Anti-α tubulin is shown as a reference indicator. **(B)** Summary of Western blot data from four experiments with HEK-293 control cells and cells exposed to 5 mM 4-PB. Upon exposure to 5 mM 4-PB Cx43 expression increased to 166 ± 11% which was statistically significant (*p* = 0.029) in comparison to the control cells (0 mM 4-PB) where Cx43 expression was assumed to be 100%.

The gap junction protein Cx43 was detected in cell cultures by Western blot analysis using a commercially available, polyclonal, anti-Cx43 antibody (C 6219, Sigma). 4-PB was exposed to cells in concentrations of: 0, 1, 2, and 5 mM. The observed changes in the Cx43 expression are shown in **Figure 5**. The Western blots qualitatively demonstrate that the total Cx43 content in HEK-293 and HeLa Cx43 cells increases upon exposure of cells to 4-PB as compared to cells not exposed to 4-PB. The quantification of the Western blots data shown in **Figure 5A** using ImageJ software yielded the following: the amount of Cx43 with 5 mM 4-PB exposure was ~70% greater in HEK-293 cells and ~40% greater in HeLa Cx43 cells in comparison to control cells. Anti-α tubulin is shown, as a reference indicating that 4-PB was the modulated variable in each of the dose experiments. Both cell types showed increased expression of Cx43 with 4-PB. The data with 5 mM 4-PB from four different experiments with HEK-293 cells are summarized in **Figure 5B**. The 4-PB treated cells exhibited statistical significant increase (*p* = 0.029) in the Cx43 expression in comparison to the control cells (166 ± 11 versus 100%, respectively).

These data are consistent with previously published studies showing 4-PB increase the expression of Cx43 and manifest in Western blot (Asklund et al., 2004; Khan et al., 2007; Dovzhanskiy et al., 2012).

## DISCUSSION

### GAP JUNCTION COMMUNICATION

The increased rate of LY dye transfer and junctional conductance seen in cells exposed to 5 mM 4-PB for 24 h indicates that there are more Cx43 gap junction channels functioning as a consequence of increased connexin expression. The conclusion that 4-PB increased expression and subsequently resulted in more functional channels is supported by the fact the single channel unitary conductance is unaffected by 4-PB along with no apparent change in open probability (Brink et al., 1996). Consistent with this conclusion is the increased expression of Cx43 as demonstrated by Western blot analysis (Asklund et al., 2004; Khan et al., 2007). The data support the notion that 4-PB enhances transcription of Cx43 mRNA, which ultimately results in an increase in the number of active channels.

However, the 4-PB induced Cx43 expression or protein abundance and functional coupling do not necessarily follow a one to one relationship. In fact previous studies have provided evidence that expression exceeds the number of functional channels (Asklund et al., 2004; Khan et al., 2007). Another confounding factor is the number of functioning channels within a junctional plaque. Bukauskas et al. (2000) demonstrated that only a small fraction (~10%) of channels function at any instant in time with a given plaque.

Histone deacetylase inhibitors are potent regulators of gene expression through their effect on the acetylation of core histones (Asklund et al., 2004). Comparison of **Figures 2A, B** indicates that cells exposed to 5 mM 4-PB allowed for greater dye transfer across the three cell liner arrays so that the cell furthest away from the source cell was able to obtain a much greater level of LY dye over a 10-min time interval. There was a marked difference in dye distribution among the three adjacent cells when comparing the control and 5 mM 4-PB groups. In the control cells, less dye was able to diffuse into the second and third cells. This is opposed to the cells exposed to 5 mM 4-PB, which exhibited a much more even distribution of dye across all three of the adjacent cells. This is consistent with a 4-PB induced increase in gap junctions resulting in a more effective diffusive pathway. In other words an increased number of Cx43 gap junction channels allows for better dye transfer between each of the cells. In the control cells, fewer Cx43 gap junction channels caused the dye to pool mostly in the first cell with a decreased transfer between cells. Hence, 4-PB was able to enhance functional intercellular communication. This finding has several implications, as LY can be substituted for any number of different drugs or second messenger molecules.

### CONNEXINS AND CARDIAC DYSFUNCTION

Currently, VT and ventricular fibrillation (VF) are two of the leading causes of death in the United States (Xing et al., 2003). Previous studies have provided evidence that abnormal functioning of gap junctions in cardiac tissue may play an important role in the induction of both VF and VT (Xing et al., 2003). In the heart, normal cardiac function relies on the electrical syncytium between adjacent cells in the myocardium. The spread of an electrical impulse is highly coordinated and any blockage that disrupts cell-to-cell communication has immediate and harmful consequences (Xing et al., 2003). A period of acute ischemia causes gap junction channels to close, leading to an uncoupling of all adjacent cells. Similarly, a prolonged period of ischemia is correlated to a non-uniform down regulation of Cx43 (Xing et al., 2003). The closure of gap junction channels combined with a decrease in Cx43 translation leaves the heart particularly susceptible to reentrant ventricular circuits and subsequently, VT or VF. The disruption of impulse propagation through the ventricular myocardium causes an irregular contraction of the ventricles. Instead of the action potential traveling normally throughout the ventricle, a decrease in gap junctions causes the electrical signal to propagate more slowly allowing for reentrant arrhythmias. This causes sporadic contraction of different parts of the ventricle known as VF. The incidence of VF has been directly related to decreased connexin levels (Xing et al., 2003). Because the drug 4-PB can induce connexin mRNA transcription, it is possible that the drug could effectively trigger renewed channel formation reduced by cardiac ischemia. Thus, the findings in this study suggest that drugs such as 4-PB which enhance gap junction coupling, might be implicated in the prevention of ischemia induced VT or VF as earlier has been shown with the anti-arrhythmic peptide ZP123 (Xing et al., 2003). In addition, another related study has shown that 4-PB significantly enhanced coupling and action potential propagation between rodent cardiomyocytes (Jia et al., 2012) suggesting that cardiac cells may be responsive to HDAC inhibitors and, hence potentially useful as a cardiac therapy.

However, it remains to be seen whether drugs like 4-PB will be effective in preventing ischemia induced cardiac maladies. Yet more thorough studies have to be done in arrhythmia models to test 4-PB potential to act as anti-arrhythmic gap junction modulator similar to anti-arrhythmic peptides AAP10 and ZP123 (Dhein et al., 2003).

## Conflict of Interest Statement

The authors declare that the research was conducted in the absence of any commercial or financial relationships that could be construed as a potential conflict of interest.
